# Probiotics ameliorate *H. pylori*-associated gastric β-catenin and COX-2 carcinogenesis signaling by regulating miR-185

**DOI:** 10.1186/s12929-025-01149-3

**Published:** 2025-06-03

**Authors:** Yao-Jong Yang, Chung-Tai Wu, Hsiu-Chi Cheng, Wei-Ying Chen, Joseph T. Tseng, Wei-Lun Chang, Bor-Shyang Sheu

**Affiliations:** 1 Department of Pediatrics, National Cheng Kung University Hospital, College of Medicine, National Cheng Kung University, #138 Sheng Li Road, Tainan, 70428 Taiwan; 2 Institute of Clinical Medicine, National Cheng Kung University Hospital, College of Medicine, National Cheng Kung University, Tainan, Taiwan; 3 Department of Internal Medicine, National Cheng Kung University Hospital, College of Medicine, National Cheng Kung University, Tainan, Taiwan; 4 College of Life Science and Medicine, National Tsing Hua University, Hsinchu, Taiwan

**Keywords:** Probiotics, MiR-185, *H. pylori*, Carcinogenesis, COX-2, Wnt/β-catenin

## Abstract

**Background:**

This study aimed to investigate whether probiotics can ameliorate the *H. pylori*-induced Wnt/β-catenin-related COX-2 carcinogenesis signaling pathway by regulating the expression of microRNAs (miRNAs).

**Methods:**

An *H. pylori* isolate and GES-1 cells were used to establish a COX-2-associated carcinogenesis axis. Western blot analysis was conducted to investigate Wnt/β-catenin and COX-2 signaling. Next-generation sequencing and DIANA Tools identified significant differences in miRNA expressions. The probiotics *Lactobacillus acidophilus* and *Bifidobacterium lactis* were used to study anti-carcinogenesis effects in GES-1 and miRNA-transfected GES-1 cells. The *H. pylori*-infected patients with intestinal metaplasia (IM) were randomly allocated into probiotic treatment or not after successful eradication, the IM regression was assessed by the 2nd esophagogastroduodenoscopy one year after treatment.

**Results:**

Pretreatment with probiotics significantly reduced *H. pylori*-induced nuclear β-catenin phosphorylation and COX-2 levels in GES-1 cells. Among 9 significantly altered miRNAs, miR-185 was the only miRNA targeting the Wnt/β-catenin signaling pathway. *H. pylori* increased miR-185 expression and upregulated COX-2 carcinogenesis through the Wnt/β-catenin pathway, but not the JAK2/STAT3 pathway. *B. lactis* ameliorated *H. pylori*-induced miR-185 expression and nuclear β-catenin/COX-2 signaling in a dose-dependent manner. In the 6-month probiotic-treated patients had a significantly higher IM regression rate than controls (intention-to-treat: 37.5 vs 11.5%, OR: 4.60, 95% CI: 1.134–18.65, *p* = 0.025; per-protocol: 46.2 vs 17.6%, OR: 4.00, 95% CI: 0.923–17.33, *p* = 0.055). Patients without IM regression had significantly higher miR-185 levels in follow-up biopsies (*p* < 0.01).

**Conclusions:**

Pretreatment with *B. lactis* ameliorated the *H. pylori*-induced COX-2 carcinogenesis pathway by reducing miR-185 expression, which targets Wnt/β-catenin signaling. (ClinicalTrials.gov, NCT05544396).

**Supplementary Information:**

The online version contains supplementary material available at 10.1186/s12929-025-01149-3.

## Background

*H. pylori* plays a major role in the development of human gastric cancer [1]. Both chronic atrophic gastritis and intestinal metaplasia (IM) are considered to be precancerous lesions of the stomach [2]. *H. pylori* elimination is the most promising strategy to reduce the incidence of gastric cancer [3], however successful eradication may only have a minor effect on IM regression [4]. Therefore, the Maastricht V/Florence Consensus suggested that a more effective strategy to reduce the risk of gastric cancer is to implement eradication treatment before the development of precancerous lesions [3]. Although it has not been definitively shown that *H. pylori* eradication can completely abolish gastric cancer, interventions to arrest or reverse the carcinogenetic axis after *H. pylori* eradication deserve deeper investigation.

Both canonical IL-11/STAT3 and Wnt/β-catenin pathways have been shown to enhance cyclooxygenase-2 (COX-2) transcriptional activity and to be involved in gastric carcinogenesis after *H. pylori* infection [5, 6]. Xiong et al. [5] demonstrated that *H. pylori* infection rapidly triggered STAT3 signaling and induced STAT3-dependent COX-2 expression, and Zhao et al. [7] found that successful *H. pylori* eradiation could ameliorate the p-STAT3 expression in patients with IM. Moreover, *H. pylori* has been shown to induce the Wnt/β-catenin pathway by upregulating c-Met and EGFR activators and downregulating trefoil factor 1 (TFF1) and Runt-related transcription factor 3 (RUNX3) suppressors in vivo and in vitro [8–12]. Therefore, in addition to early eradication of *H. pylori* in patients with IM, strategies to counteract both carcinogenetic pathway may reduce the rate of gastric cancer development.

Our previous studies showed that *H. pylori* eradication plus celecoxib (a COX-2 inhibitor) therapy resulted in IM regression, and that this effect was dependent on nuclear β-catenin and COX-2 expressions before treatment [13–15]. Based on these findings, we hypothesized that strategies to downregulate COX-2 expression may facilitate IM regression after the eradication of *H. pylori*. In addition, we previously found that *Lactobacillus acidophilus* ameliorated *H. pylori*-induced gastric inflammation by inactivating the Smad7 and NFκB pathways [16]. Moreover, a clinical trial also showed that probiotic-containing yogurt ingestion could significantly alter the gut microbiota and reduce the serum IL-6 level in *H. pylori*-infected children [17]. Furthermore, several *H. pylori*- and chemical-induced gastro-colitis mice models showed that probiotics could significantly promote the downregulation of COX-2 signaling [18, 19]. Taken together, these findings imply that probiotics may have an anti-COX-2 carcinogenesis effect in the *H. pylori*-related gastric carcinogenetic process.

MicroRNAs (miRNAs) are a class of widespread non-coding RNAs, many of which are involved in cell growth, differentiation and carcinogenesis. Previous studies have shown that several miRNAs are negatively or positively involved in gastric carcinogenesis in both metaplasia and cancer development [20–25], and that some of them interact with the Wnt/β-catenin pathway [23–25]. Nevertheless, these studies were limited by focusing on a single miRNA or the miRNAs were detected by microarray. Therefore, further studies are needed to validate the interactions between miRNAs and Wnt/β-catenin carcinogenesis pathway using powerful tools such as next generation sequencing. Moreover, it would be interesting to investigate whether probiotics can ameliorate the *H. pylori*-related Wnt/β-catenin pathway by targeting related miRNAs. Therefore, we conducted this study to investigate these issues further.

## Methods

### Bacteria and cell lines

Human gastric epithelial cancer cell lines AGS (the Health Science Research Resources Bank, Japan), and human gastric epithelial immortalized GES-1 cells (purchased from Prof. Jia, Shandong University, China) were cultured in RPMI 1640 or DMEM medium (GIBCO BRL, Grand Island, NY) supplemented with 10% FCS, 100 U penicillin per ml and 100 U streptomycin per ml in air with 5% CO_2_ at 37 °C. The cells were subcultured every second day. Prior to the bacterial infection study, the cells were incubated in antibiotic-free RPMI 1640 medium containing 10% FCS overnight at 37 °C in 5% CO_2_.

*H. pylori* strain HP238 isolated from a Taiwanese patient with gastric mucosa-associated lymphoid tissue lymphoma was subcultured. This strain has been shown to express CagA, VacA, and BabA proteins in previous studies [26]. The other two strains, *H. pylori* 26,695 (a strain isolated from a patient with chronic gastritis) and HP1031 (a clinical strain isolated from a patient with adenocarcinoma) were used for test as well. *H. pylori* cultures were maintained on a Brucella agar plate containing 10% horse serum and incubated under microaerophilic conditions (10% CO_2_, 5% O_2_ and 85% N_2_) for 24–48 h before use. After thawing, the second to fifth generations of the strain were used. The probiotic strains *Lactobacillus acidophilus* and *Bifidobacterium lactis* (LA5® & BB12®, originating from Chr. Hansen, Denmark, provided by the President Corp., Tainan, Taiwan) contained in AB-powder were used. The bacteria were maintained on a Brucella agar, incubated in anaerobic conditions, and then harvested and suspended in phosphate-buffered saline (PBS) before infection. The viable densities of *H. pylori*, *L. acidophilus* and *B. latis* were 1 × 10^8^ bacteria/ml at an OD of 1, respectively.

### Co-culture with bacteria and cells

Cells were prepared by seeding 1 × 10^6^ cells on plates. After overnight incubation, the medium was replaced with fresh medium without antibiotics. The bacteria were harvested from the plates by suspension in 2 ml serum-free medium and washing twice with PBS. After centrifugation at 4000 rpm for 5 min, the bacteria were resuspended in 2 ml PBS and added immediately to cell culture plates at a multiplicity of infection of 100 for various time periods. To assess whether the probiotics could inhibit *H. pylori*-induced oncogenesis cascades, the cells were pretreated with *L. acidophilus, B. lactis*, or both for 2 h, and then washed with PBS and infected with *H. pylori* for an optimal period. The co-cultures were incubated in air with 5% CO_2_ at 37 °C.

### Enzyme-linked immunosorbent assay for IL-6, IL-11, and IL-8

The bacteria were cultured with cells in different in vitro tests. Each final culture supernatant was centrifuged at 12,000 rpm for 5 min to remove bacteria and cell debris. The concentrations of IL-6, IL-11, and IL-8 were measured using a DuoSet ELISA Development kit (R&D Systems, Minneapolis, MN) according to the manufacturer’s instructions. The absorbance of each microplate was read on a spectrophotometer using 450 nm as the primary wave length and 570 nm as the reference wave length.

### Western blotting for JAK2/STAT3 and Wnt/β-catenin-induced COX-2 cascades

The cytoplasmic and nuclear extracts from the bacteria and cell co-incubations were washed with ice-cold PBS and lysed in a 0.5 ml/well lysis buffer (150 mmol/l NaCl, 20 mmol/l Tris, pH 7.5, 0.1% Triton X-100, 1 mmol/l phenylmethylsulfonyl fluoride [PMSF] and 10 μg/ml aprotonin) as previously described [15]. Protein concentrations in the lysates were determined using a Pierce BCA Protein Assay Kit (Thermo Scientific, USA). Twenty μg protein per lane was then size-fractionated into a denaturing, non-reducing 10% polyacrylamide minigel and electrophoretically transferred to polyvinylidene fluoride (PVDF) membranes (0.45-μm pore size) (Millpore Corporation, USA). Specific proteins were detected using commercial antibodies as the primary antibodies (1:500~1:1000), and peroxidase-conjugated anti-rabbit IgG, anti-mouse IgG (1:10,000) as the secondary antibodies. Specifically bound peroxidase was detected using Chemiluminescent HRP Substrate (ECL system, Millpore Corporation, USA) and then exposed to chemiluminescence (Invitrogen, USA) for 10–30 s.

### Probiotic administration in *H. pylori*-infected patients with IM after eradication

Patients aged >18 years who underwent esophagogastroduodenoscopy (EGD) for various gastrointestinal diseases and were diagnosed with *H. pylori* infection with histological IM were enrolled after obtaining consent. The Ethics Committee of our Institutional Review Board approved this study (A-BR-104-091 & A-BR-106-085). The patients received eradication therapy, and successful eradication of *H. pylori* was confirmed by ^13^C-UBT at 4–6 weeks after completing eradication therapy. The patients were then randomly allocated to the treatment or control group (Suppl. Figure 3) by an assistant or host draws lots (group 1 and group 2). Patients in the treatment group received AB powder (the President Corp., Tainan, Taiwan) 1 pack twice daily for 6 months. The powder contained at least 5 × 10^9^ live organisms (*Lactobacillus acidophilus* LA-5®, *Bifidobacterium lactis* BB-12®, Chr. Hansen, Hoersholm, Denmark and *Lactobacillus acidophilus* La-14, International Flavors & Fragrances, Sydney, Australia). Repeat EGD was performed 1 year after allocation to evaluate the effects of probiotics on IM regression.

### Sample size and statistical analysis

We estimated that there would be a 25% improvement in IM regression rate between patients with probiotic treatment and control on previous studies [13, 14]. Therefore, the estimated regression rate of the patients with successful eradication therapy in the control group was set as 20%.^13^ With a two-sided α value of 0.05 and a power of 90% (β = 0.1), the total number of patients required was 72 using the Power and Sample Size Calculation software. The Student’s t test was used to examine differences in parametric variables. Pearson’s χ^2^ test was used to examine differences in IM regression rate between the treatment and control groups.

## Results

### *H. pylori* induced cytokine expressions in primary (GES-1) and cancerous (AGS, MKN45, and SUN1) gastric cells

**Fig. 1:**
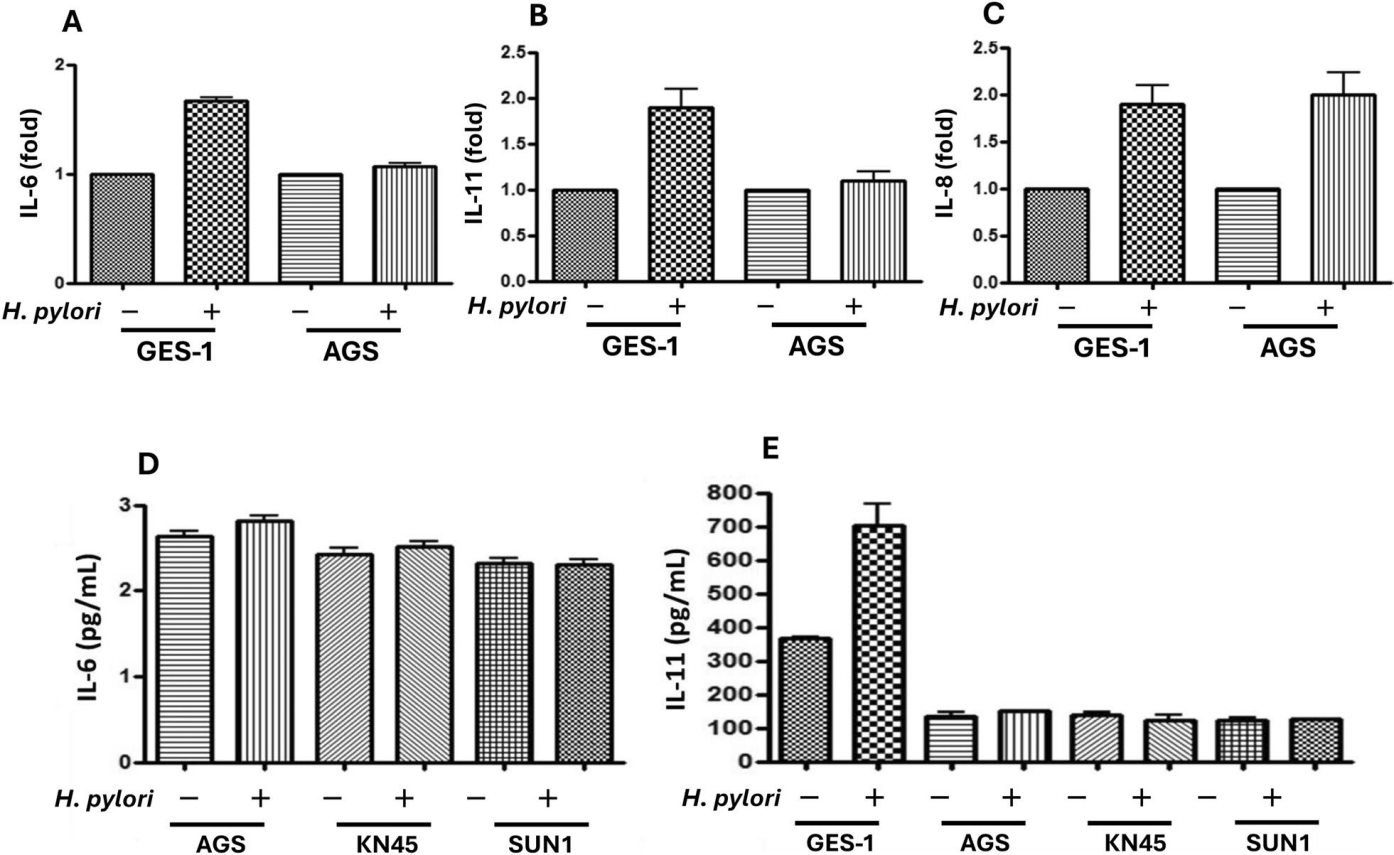
The levels of IL-6, IL-11, and IL-8 after *H. pylori* infection in both cancerous (AGS, MKN45, and SUN1) and primary GES-1 cells. The cells were seeded in one layer on the plates. *H. pylori* and PBS (control) were co-incubated with the cells for 24 h, and the supernatant was collected and evaluated for IL-6 (**a** and **d**), IL-11 (**b** and **e**), and IL-8 (**c**) levels by ELISA

Figure 1 shows the levels of IL-6, IL-11, and IL-8 after *H. pylori* 238 infection in both primary and cancerous gastric cells. In primary gastric GES-1 cells, *H. pylori* infection increased IL-6, IL-11, and IL-8 expressions. However, *H. pylori* infection induced only IL-8 but not IL-6 and IL-11 levels in the cancerous AGS cells (Fig. 1A–C). We further confirmed the differences in cytokine induction between primary and cancerous cells, and showed that *H. pylori* could not induce IL-6/IL-11 expressions after 24 h of incubation with both MKN45 and SUN1 cancerous cells (Fig. 1D, E).

### *H. pylori* induced nuclear β-catenin/COX-2 expressions in both GES-1 and AGS cells

**Fig. 2:**
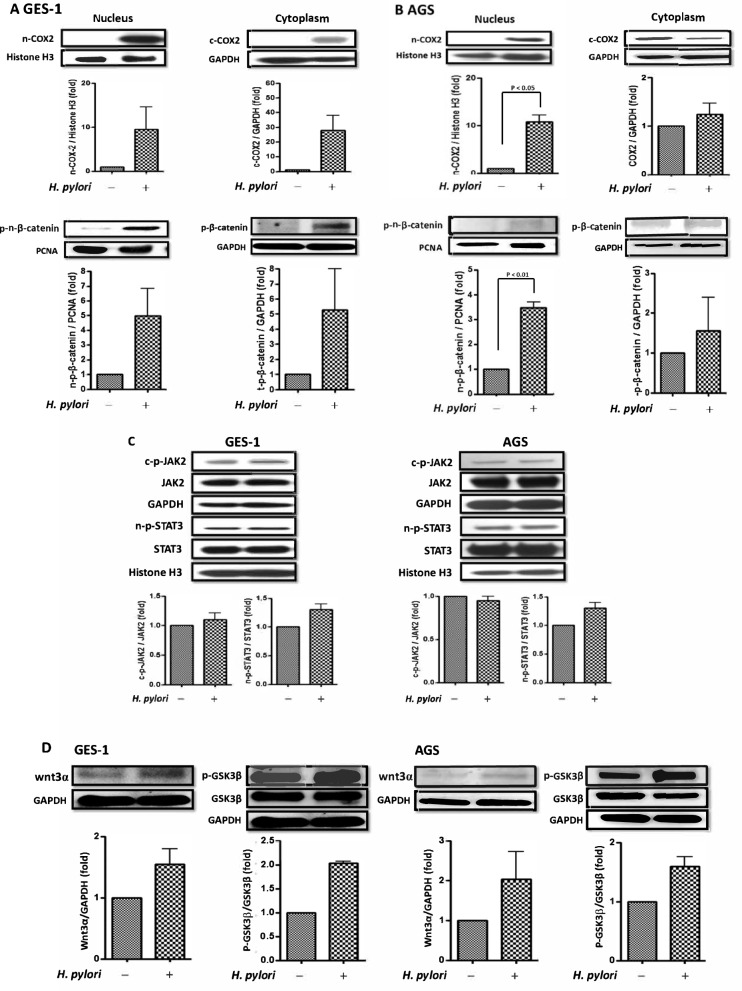
*H. pylori* infection induced β-catenin/COX-2 expressions in GES-1 and AGS cells. **A** The nuclear and cytoplasmic protein levels (fold) of GES-1 cells incubated with *H. pylori* or PBS (control) for 24 h. **B** The nuclear and cytoplasmic protein levels (fold) of AGS cells incubated with *H. pylori* or PBS (control) for 24 h. **C** The protein levels (fold) of cytoplasmic JAK2 and nuclear p-STAT3 in both GES-1 and AGS cells after co-culture with *H. pylori* or PBS for 24 h. **D** The protein levels (fold) of Wnt3α and p-GSK3β of GES-1 and AGS cells co-cultured with *H. pylori* or PBS for 24 h

The *H. pylori*-induced β-catenin/COX-2 expressions in GES-1 and AGS cells are shown in Fig. 2. Although *H. pylori* activated IL-6 and IL-11 only in GES-1 but not in AGS cells, *H. pylori* infection upregulated both nuclear and cytoplasmic β-catenin/COX-2 expressions in GES-1 cells (Fig. 2A). In contrast, the β-catenin/COX-2 induction by *H. pylori* was obvious in the nucleus but weak in the cytoplasm in AGS cells (Fig. 2B). We further tested the two major upstream signaling pathways (JAK2/STAT3 and WNT/β-catenin) of COX-2 activation after *H. pylori* infection in both GES-1 and AGS cells. The results showed that JAK2/STAT3 signaling was weakly upregulated after *H. pylori* infection in the nucleus and cytoplasm in both GES-1 and AGS cells (Fig. 2C). In contrast, *H. pylori* infection obviously activated nuclear phosphorylated β-catenin level (Fig. 2A, B) by upregulating Wnt3α/GSK3β in both GES-1 and AGS cells (Fig. 2D).

### Pretreatment with probiotics ameliorated *H. pylori*-induced COX-2 expression

**Fig. 3:**
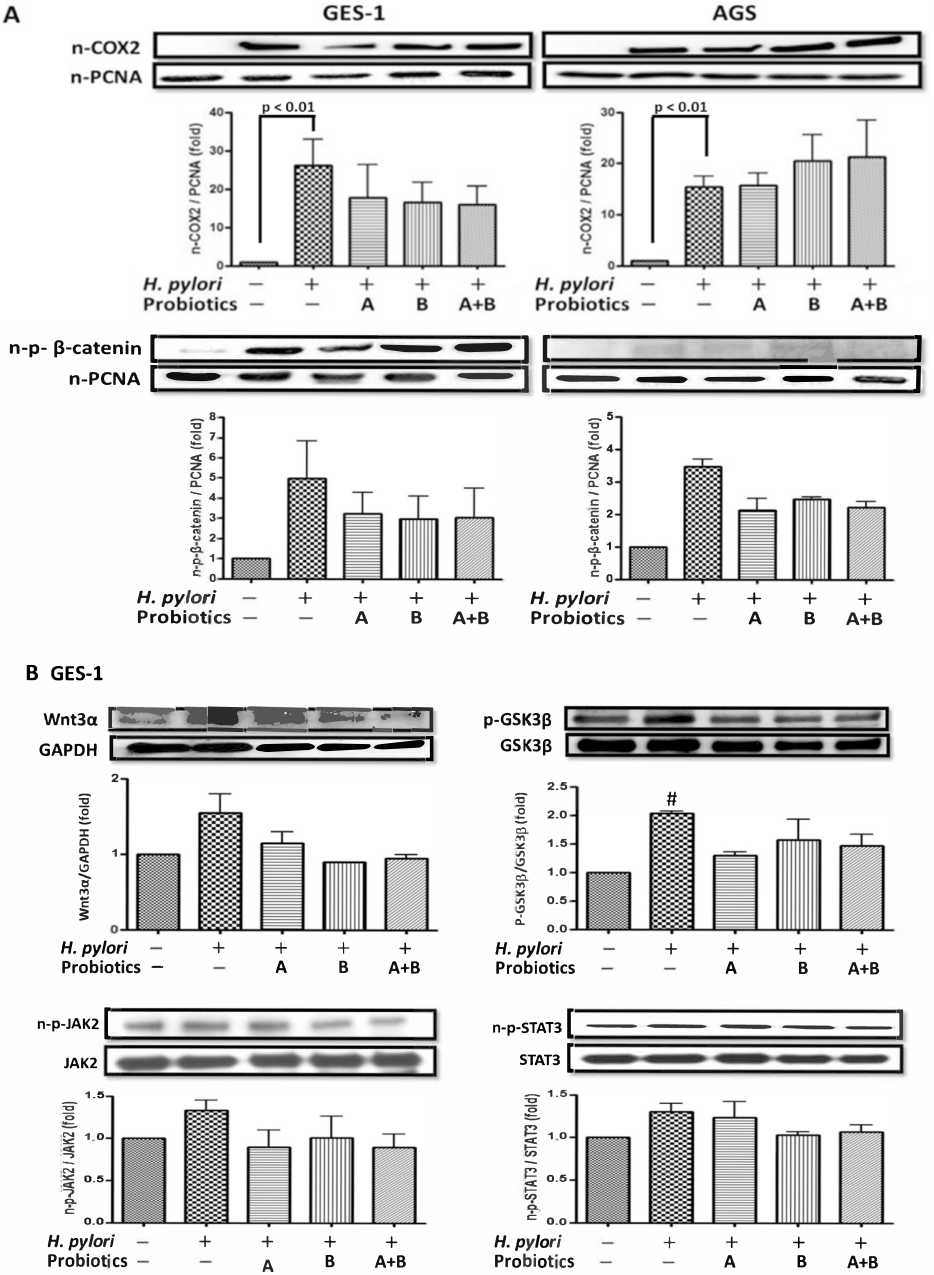
Pretreatment with probiotics (A. *L. acidophilus*, B. *B. lactis*, A + B. mixture of A and B) reduced nuclear phosphorylated β-catenin and COX-2 which were upregulated by *H. pylori*. The cells were pretreated with probiotics (MOI 100) for 4 h. The cells were washed with PBS 3 times and then co-cultured with *H. pylori* for 24 h. The nuclear protein was harvested for **A** COX-2 and β-catenin and **B** Wnt3α/p-GSK3β and JAK2/nuclear p-STAT3

Due to the obvious *H. pylori*-induced COX-2 expression in the nucleus of GES-1 and AGS cells, we further tested the protective effects of probiotics on nuclear STAT3/COX-2 and β-catenin/COX-2 signaling pathways (Fig. 3). Figure 3A shows that pretreatment with probiotics (*L. acidophilus*, *B. lactis*, and a mixture of both) obviously ameliorated the *H. pylori*-induced nuclear COX-2 expression in primary GES-1 cells. However, in AGS cells, there was no benefit in counteracting *H. pylori*-induced nuclear COX-2. Therefore, we further confirmed that the anti-COX-2 effect of probiotics in GES-1 cells was through the inhibition of Wnt/β-catenin signaling rather than the JAK2/STAT3 pathway (Fig. 3B). Moreover, the anti-COX-2 effect of *B. lactis* pretreatment seemed to be better than that of *L. acidophilus* pretreatment.

### Probiotics counteracted *H. pylori*-induced alterations of miRNAs in GES-1 cells

The total RNA samples collected from cell lysates of GES-1 cells infected with *H. pylori*, *H. pylori* with probiotic pretreatment, and controls were sent for next generation sequencing analysis. Significant changes were found in 9 miRNAs, which were upregulated (miR-190a, miR-501, and miR-4798) or downregulated (miR-27a, miR-30c-2, miR-93, miR-140, miR-185, and miR-1307) by *H. pylori* and rescued by probiotics at a level >20% change (Table 1). Among these candidate miRNAs, only miR-185 was predicted to involve the Wnt/β-catenin signaling pathway according to DIANA Tools (https://diana.e-ce.uth.gr/home). Therefore, we further tested the role of miR-185 in probiotics counteracting the *H. pylori*-related Wnt/β-catenin/COX-2 signaling carcinogenesis pathway.

**Table 1:** Candidate miRNAs in GES-1 cells induced by *H. pylori* and reversed by probiotics with at least 20% change

miRNA	Normalized expression values				Fold change				Probiotic regulation
	Control	*H. pylori*	(L + B)	(L + B) + *H. pylori*	*H. pylori*/control	(L + B)/control	(L + B) + *H. pylori*/control	(L + B) + *H. pylori*/*H. pylori*	
mir-1307	2968.97	3663.41	3171.97	2474.78	1.23	1.07	0.83	0.68	Down-regulation
mir-93	2170.11	2800.01	2176.75	2161.47	1.29	1.00	1.00	0.77	Down-regulation
mir-185	632.07	840.31	684.50	542.13	1.33	1.08	0.86	0.65	Down-regulation
mir-27a	2073.91	2927.01	2249.79	1810.15	1.41	1.08	0.87	0.62	Down-regulation
mir-30c-2	333.59	477.38	374.54	367.52	1.43	1.12	1.10	0.77	Down-regulation
mir-140	591.97	866.91	661.34	575.22	1.46	1.12	0.97	0.66	Down-regulation
mir-501	11.77	5.02	12.55	10.56	0.43	1.07	0.90	2.10	Upregulation
mir-190a	4.52	2.01	4.18	4.22	0.44	0.93	0.93	2.10	Upregulation
mir-4798	2.16	1.00	2.15	2.11	0.46	0.99	0.98	2.10	Upregulation

(L + B): probiotics containing *L. acidophilus* and *B. latis*

### *H. pylori* induced miR-185 and downstream β-catenin/COX-2 signaling

**Fig. 4:**
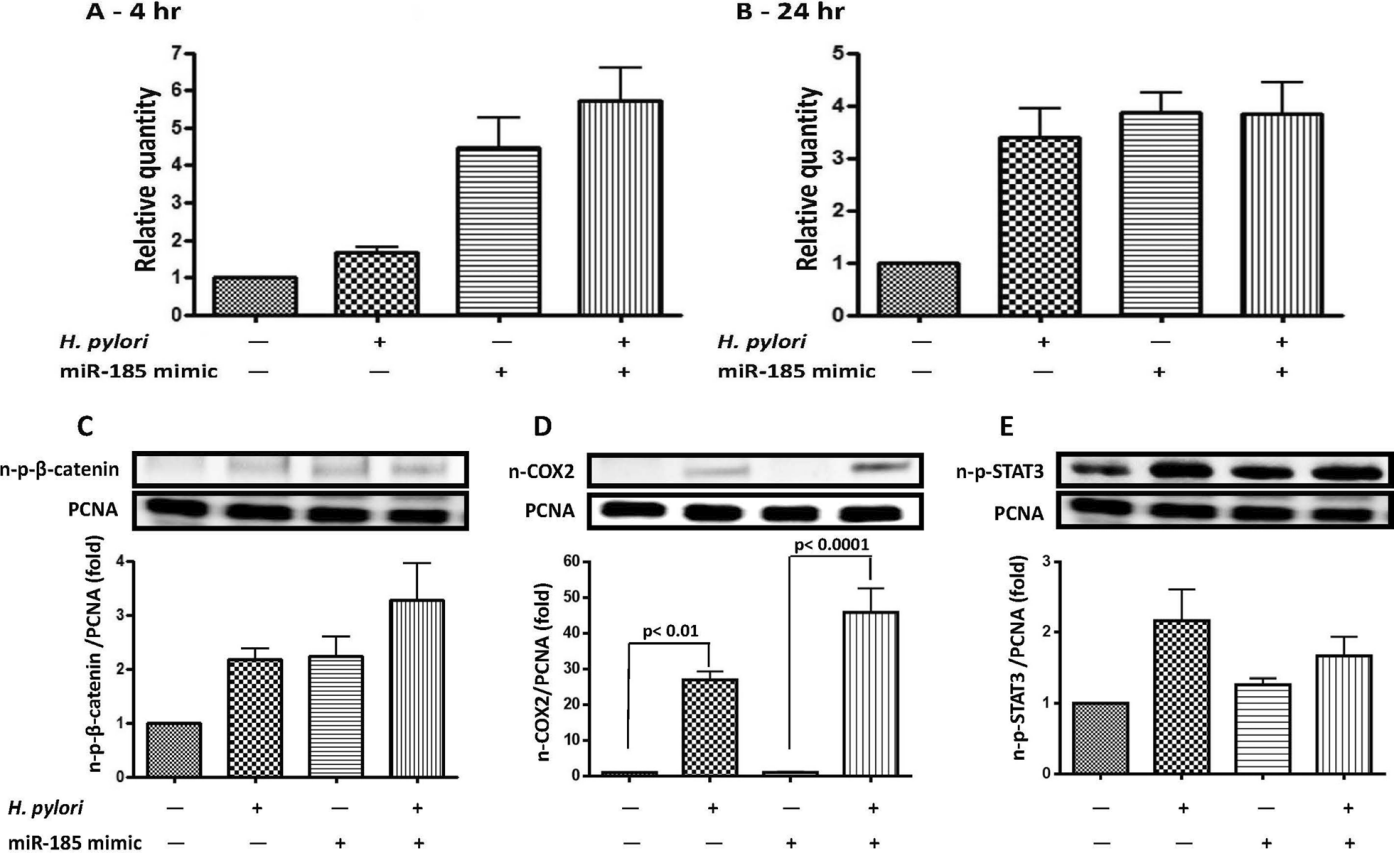
The miR-185 transcription level in GES-1 and miR-185-overexpressing GES-1 (miR-185 mimic) cells after *H. pylori* infection. The relative quantity of miR-185 was measured by real-time PCR at **A** 4 h and **B** 24 h. The protein levels of nuclear p-β-catenin **C**, p-STAT3 **E**, and COX-2 **D** in GES-1 and miR-185-overexpressing GES-1 (miR-185 mimic) cells after *H. pylori* infection

To investigate the role of miR-185 on regulating β-catenin/COX-2 signaling after *H. pylori* infection, we conducted miR-185 mimic (GES-1^miR−185+^) and inhibitor (GES-1^miR−185−^) transfection in the GES-1 cells. The relative quantities of miR-185 and β-catenin/COX-2 expressions in GES-1^miR−185+^ and GES-1^miR−185−^ cells are shown in Supplementary Fig. 1, respectively. The relative quantity of miR-185 in GES-1^miR−185+^ cells significantly increased after 72 h of incubation (Suppl. Figure 1 A). However, even with a higher dose of transfection, there was no obvious decrease in miR-185 in GES-1^miR−185−^ cells after 72 h of incubation (Suppl. Figure 1B), although translation levels of downstream β-catenin phosphorylation and COX-2 were achieved (Suppl. Figure 1 C & 1D). Interestingly, in miR-185-overexpressing cells, the ability of *H. pylori* to induce an increase in miR-185 expression was diminished (Fig. 4A, B). *H. pylori* infection induced a higher nuclear β-catenin/COX-2 expression in GES-1^miR−185+^ than in GES-1 cells (Fig. 4C, D). In contrast, miR-185 overexpression in GES-1^miR−185+^ cells did not affect nuclear STAT3 phosphorylation after *H. pylori* infection (Fig. 4E).

### Pretreatment with probiotics counteracted *H. pylori*-induced miR-185 expression in GES-1 cells but had a smaller effect in GES-1^miR−185+^ cells

**Fig. 5:**
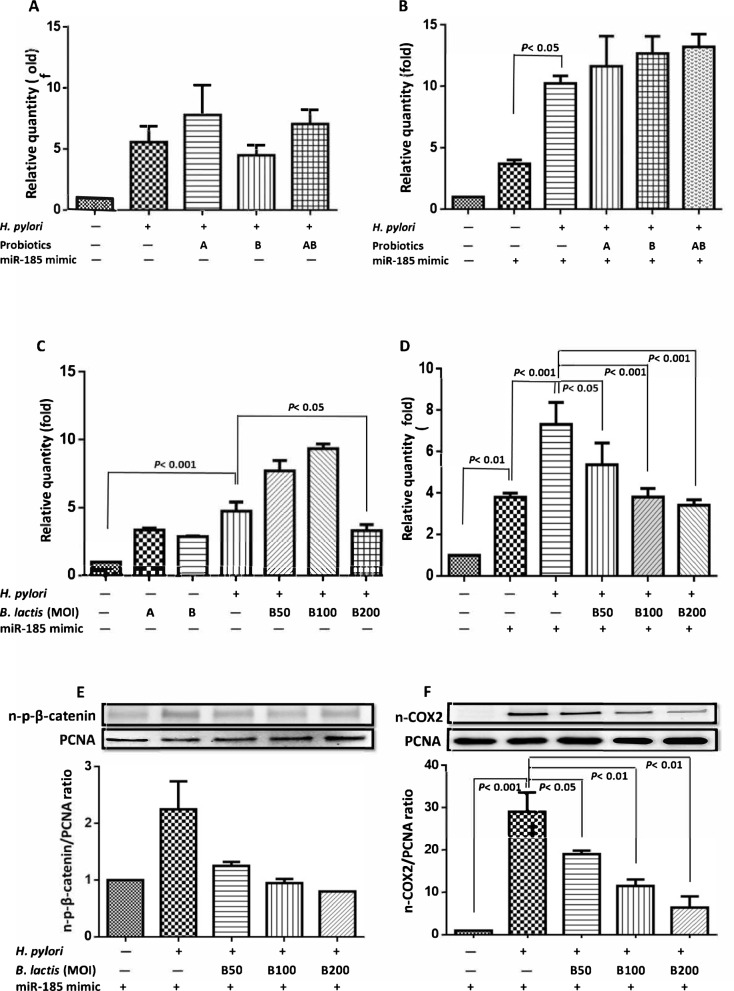
Effect of pretreatment with. probiotics (A. *L. acidophilus*, B. *B. lactis*, A + B. mixture of A and B) on the relative quantity of miR-185 in **A** GES-1 and **B** miR-185-overexpressing GES-1 (miR-185 mimic) cells after *H. pylori* infection. The pretreatment with differential doses of *B. lactis* (B50: MOI 50, B100: MOI 100, and B200: MOI200) on the relative quantity of miR-185 in **C** GES-1 and **D** miR-185 mimic cells and nuclear p-β-catenin and COX-2 in **E** GES-1 and **F** miR-185 mimic cells after *H. pylori* infection

We further tested whether probiotic treatment inhibited *H. pylori*-induced β-catenin/COX-2 carcinogenesis signaling by regulating miR-185 (Fig. 5). Figure 5A and B show the relative quantities of miR-185 in GES-1^miR−185+^ and GES-1 cells with and without probiotic treatment after *H. pylori* infection. Pretreatment with *B. lactis* (MOI 100) partially reduced miR-185 production in GES-1 cells after *H. pylori* infection (Fig. 5A). However, pretreatment with *L. acidophilus* did not. In contrast, in *H. pylori*-infected GES-1^miR−185+^ cells, pretreatment with *L. acidophilus*, *B. lactis*, and a mixture of both did not reduce the relative miR-185 levels (Fig. 5B). Because *B. lactis* had a better anti-miR-185 effect than *L. acidophilus*, we further investigated the dose–effect of *B. lactis* in counteracting *H. pylori*-induced miR-185 expression. The results showed that a higher dose of *B. lactis* significantly ameliorated the relative quantity of miR-185 in GES-1 (Fig. 5C) and GES-1^miR−185+^ (Fig. 5D) cells after *H. pylori* infection. In addition, pretreatment with *B. lactis* significantly reduced nuclear β-catenin/COX-2 expressions in GES-1^miR−185+^ cells in a dose-dependent manner (Fig. 5E, F).

### Probiotics promoted the IM regression rate in *H. pylori*-infected patients after eradication in a clinical trial

Supplement Fig. 2 shows the study flow diagram and the number of patients in the clinical trial (NCT05544396). A total of 2037 dyspeptic patients who received EGD and biopsies were enrolled, and the histopathology was evaluated. Five hundred and ninety-two (29.1%) patients were diagnosed with *H. pylori* infection by rapid urea test and histology. Of them, 165 (27.9%) had IM. After excluding 107 patients (30 who were unwilling to enter the study, 61 who previously failed eradication, 1 with mania disease, 7 with gastric cancer, 1 who died of causes unrelated to gastric disease, and 7 with eradication failure), 58 patients (32 in the probiotic group, 26 controls) were randomly allocated to receive probiotics or not. The demographic data of allocated subjects was shown in the Supplementary Table 1. There were no significant differences of age, gender, and scores of IM between groups. After 6 months of probiotic treatment, the patients in the probiotic treatment group had a significantly higher IM regression rate than the controls in intention-to-treat (ITT) analysis (37.5 vs 11.5%, OR: 4.60, 95% CI: 1.134–18.65, *P* = 0.025) and borderline significantly higher IM regression rate in per-protocol (PP) analysis (46.2 vs 17.6%, OR: 4.00, 95% CI: 0.923–17.33, *P* = 0.055). None of the participants reported serious complications related to the probiotic treatment.

### Patients without IM regression had a significantly higher gastric miR-185 expression 1 year after *H. pylori* eradication

**Fig. 6:**
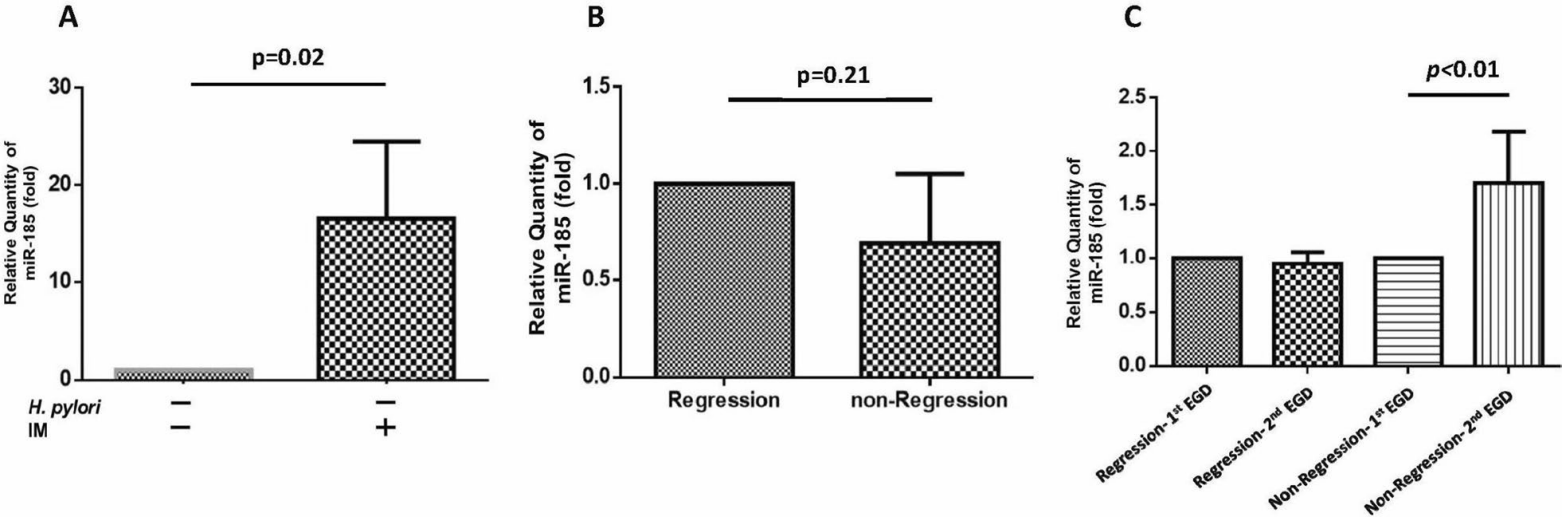
The mean relative transcription levels of miR-185 in antrum biopsies between **A** IM and non-IM patients without *H. pylori* infection. **B** The mean miR-185 expression in antrum biopsies of *H. pylori*-infected patients with IM who completed a 6-month course of probiotic treatment 1 year after eradication therapy. **C** The mean miR-185 transcription levels stratified by regression and non-regression of IM at the second EGD. EGD, esophagogastroduodenoscopy; IM, intestinal metaplasia

To investigate the correlation between gastric miR-185 expression and the presence of IM, we randomly invited 8 dyspeptic patients without *H. pylori* infection who had undergone EGD to provide additional antral tissues for miR-185 examinations by real-time PCR. Three of them were found to have IM and 5 did not on histologic examination. Figure 6 shows the mean relative transcription levels of miR-185 in gastric tissues between groups. In the patients without *H. pylori* infection, the antral biopsies from the patients with IM had a significantly higher miR-185 level than those without IM (Fig. 6A). We also randomly invited 8 patients in the probiotic groups to provide an additional piece of antral biopsy when performing the first and second EGDs. The results showed that 5 (62.5%) of them had IM regression in the biopsies at the second EGD. Furthermore, the mean expression of miR-185 was not different between the patients with or without IM regression (*P* = 0.21) 1 year after the 6-month probiotic course of treatment (Fig. 6B). Figure 6C shows the mean miR-185 transcription levels stratified by the regression and non-regression of IM at the first and second EGDs. The results showed that although there was no significant reduction in miR-185 level in the patients with regression, the miR-185 level was significantly higher in the patients without IM regression.

## Discussion

This study is the first to demonstrate that probiotic treatment can counteract the *H. pylori*-induced Wnt/β-catenin-related COX-2 signaling pathway by inhibiting miR-185. *H. pylori* infection induces gastric inflammation and may consequently promote gastric carcinogenesis. *H. pylori*-induced IL-6/IL-11 driving JAK/STAT3 activation plays a major role in the development and progression of gastric cancer [5, 27–29]. In this study, we found that *H. pylori* activated IL-6/IL-11 in primary gastric epithelium GES-1 cells but not in cancerous AGS, MKN45 and SUN1 cells. These results indicate that IL-11 may serve as a driver in *H. pylori*-induced gastric carcinogenesis in primary gastric cells. However, once cancer is established, the role of *H. pylori*-induced IL-11 is no longer the major factor for cancer progression.

This study confirms again that *H. pylori* induces the COX-2 carcinogenesis process through the Wnt/β-catenin rather than JAK2/STAT3 signaling pathway in both GES-1 and AGS cells [6, 11]. However, these results are inconsistent with those reported by Piao et al. [29]. This may be due to the different strains used. In this study, we used the *H. pylori* HP238 strain isolated from a Taiwanese patient with gastric mucosa-associated lymphoid tissue lymphoma [27]. In contrast, the *H. pylori* 43,504 strain used by Piao et al. originated from an Australian patient with gastric cancer. To study whether the *H. pylori*-indued β-catenin/COX-2 signaling in GES-1 cells is strain-dependent or not, we further tested additional two strains (HP26695 and HP1031) to show all of them induced β-catenin/COX-2 carcinogenetic pathway (Suppl. Figure 4). Moreover, in our previous studies, we showed that the intensity of nuclear β-catenin expression determines the presence or lack of IM regression after *H. pylori* eradication [14, 15]. Therefore, *H. pylori* infection-induced nuclear β-catenin/COX-2 expression not only triggers the carcinogenic process in primary gastric cells but also promotes cancer proliferation and progression.

Probiotics have been shown to reduce inflammatory cytokines and NFκB in *H. pylori*-related gastric diseases [16, 17] and colitis [19]. However, whether probiotics have an anti-carcinogenesis affect has rarely been investigated. In this study, we showed that pretreatment with probiotics obviously abolished *H. pylori*-induced nuclear COX-2 expression in GES-1 cells (Fig. 3A). Interestingly, this anti-COX-2 effect was not found in AGS cells. This implies that the administration of probiotics had a beneficial effect on COX-2-related gastric carcinogenesis only in the prevention rather than treatment of gastric cancer. On the other hand, once carcinoma developed, the administration of probiotics did not affect the COX-2 oncogenic pathway. Moreover, our results also demonstrated that *B. lactis* had a stronger anti-COX-2 effect than *L. acidophilus* in the GES-1 cells. Future studies are needed to investigate the differential metabolomics between *B. lactis* and *L. acidophilus*.

We also found that the anti-COX-2 activity of *B. lactis* was partly by downregulating the JAK2/STAT3 pathway. Lee et al. reported that probiotic treatment activated the suppressor of cytokine signaling (SOCS) expression in AGS cells through STAT-1/STAT-3 activation and JAK2 inactivation [30]. Interestingly, although pretreatment with *B. lactis* and *L. acidophilus* did not reduce nuclear COX-2 in the AGS cells, both of them had a strong inhibiting effect on nuclear STAT3 phosphorylation (Suppl. Figure 3). Future studies are needed to investigate the targeted effects of probiotics on inhibiting STAT3 activation.

MicroRNAs function as tumor suppressors or enhancers by targeting the Wnt/β-catenin signaling pathway in gastric carcinogenesis [23–25, 31–34]. Qiu et al. [31] reported that the overexpression of miR-185 in MGC803 gastric cancer cells inhibited gastric oncogene TRIM29 expression and activity of Wnt/β-catenin signaling. In contrast, in the present study, we showed that the overexpression of miR-185 in GES-1 cells was upregulated by *H. pylori* and significantly reduced by probiotics, and that miR-185 was the only miRNA targeting β-catenin signaling in DIANA Tools analysis. We also showed that *H. pylori* infection-induced miR-185 targeting the β-catenin/COX-2 signaling pathway could be strengthened by miR-185 overexpression and diminished by inhibitor-transfected GES-1 cells (Fig. 4). These results imply that miR-185 is an oncogene in *H. pylori*-related gastric carcinogenesis. The possible mechanisms underlying *H. pylori*-induced miR-185 expression is unclear. Chronic infection with *H. pylori* is the strongest known risk factor for the development of gastric cancer. Shao et al. have reported that *H. pylori* increases miR-135b-5p expression by activating the NF-κB signaling pathway [35]. This finding indicates that *H. pylori* triggers the dysregulation of miRNA expression may through inducing chronic inflammatory process. The detail mechanism warrants further studies.

To study whether probiotic pretreatment could have a beneficial effect on *H. pylori*-induced miR-185 expression, we designed an in vitro experiment with GES-1 and GES-1^miR−185+^ cells with various probiotic doses. The reduction of miR-185 by *B. lactis* pretreatment is not statistically significant (Fig. 5A), yet it is considered biologically relevant. However, *B. lactis* failed to reduce miR-185 expression in overexpressed cells (Fig. 5B), this discrepancy may cause by either the miR-185 overexpression levels exceed the regulatory capacity of *B. lactis* or if other factors are at play. In dose-dependent effect of *B. lactis* on miR-185 tests showed that the administration of *B. lactis* ameliorated *H. pylori*-induced miR-185 production in GES-1 cells with an abrupt decrease miR-185 expression in a MOI200 pretreatment (Fig. 5C). Interestingly, if miR-185 was overexpressed in the GES-1^miR−185+^ cells, the effect of *B. lactis* was more significant in a dose-dependent manner (Fig. 5D). In addition, the dose-dependent effect was concomitant with a reduction in nuclear β-catenin/COX-2 levels in the GES-1^miR−185+^ cells (Fig. 5E). These results highlight the role of miR-185 in *H. pylori*-related carcinogenesis and the value of probiotics (esp. *B. lactis*) on IM regression after *H. pylori* eradication.

Based on the possible anti-carcinogenesis function of probiotics, we screened and treated a large dyspeptic population with *H. pylori* infection and IM. The results of the clinical trial showed a favorable IM regression rate in the probiotic-treated group compared to the controls. The administration of probiotics resulted in a 4–fivefold increase in the regression rate of IM compared to the controls after *H. pylori* eradication. In comparison, the IM regression rate in patients treated with celecoxib, a COX-2 inhibitor, in our previous study [14] was lower than that in the present study (ITT, 24.2 vs 37.5%; PP, 46.2 vs 28.6%). This difference may be due to the case selection, which included treatment-naïve patients in the current study compared to patients with persistent IM after treatment for 3 years in the celecoxib study. Consistent with the results of the in vitro study, the RNA level of miR-185 was significantly higher in the patients with IM than in those without IM and without *H. pylori* colonization in the gastric tissue study. In the probiotic treatment group, there was no significant difference in the tissue level of miR-185 between the first and second EGD examinations in the patients with IM regression. This indicates that gastric β-catenin/COX-2 rather than miR-185 levels could predict IM regression or not after *H. pylori* eradication [15]. However the gastric miR-185 level was significantly higher in the second EGD than the first EGD in the patients without IM regression, which implies that a higher gastric miR-185 level may be a predictive factor for IM persistence or progression in patients at risk of cancer development.

This study has some limitations. First, we did not check specific associations between different *H. pylori* or Bifidobacteria strains and miR-185/β-catenin/COX-2 pathway. Second, the mechanism underlying the anti-COX-2 effect of probiotics in AGS cells remains unclear. Third, we did not answer the inadequate reduction of RNA level in miR-185 knockdown GES-1 cells (GES-1^miR−185−^). Whether the inhibitor’s efficacy can be achieved by increasing the dose more than 30 nM or trying alternative transfection methods (e.g., different reagents or incubation times) needs further investigation. Fourth, the number of cases was relatively small and duration of follow-up was shorter in the clinical trial. The more participants and longer follow-up duration are needed in future trials.

## Conclusions

MiR-185 is an important miRNA involved in the *H. pylori*-induced β-catenin/COX-2 carcinogenesis pathway. The administration of probiotics promoted IM regression after *H. pylori* eradication by counteracting the miR-185-induced β-catenin/COX-2 axis.

## Supplementary Information

Additional file 1.

Additional file 2.

Additional file 3.

Additional file 4.

Additional file 5.

## Data Availability

The datasets supporting conclusions of this article are available from the corresponding author upon reasonable request.

## Abbreviations

COX-2: Cyclooxygenase-2
miRNAs: MicroRNAs
IM: Intestinal metaplasia
TFF1: Trefoil factor 1
RUNX3: Runt-related transcription factor 3
EGD: Esophagogastroduodenoscopy

## Declarations

### The name of Randomized Clinical Trials registry and the registration number

Name: Study on the Probiotics Regulating miRNA in *H. pylori*-induced Wnt/β-catenin Gastric Carcinogenesis. ClinicalTrials.gov ID: NCT05544396. Date of registered this trial: March 20, 2024. https://clinicaltrials.gov/study/NCT05544396.
