# Supplementary material for: Probiotics ameliorate H. pylori-associated gastric β-catenin and COX-2 carcinogenesis signaling by regulating miR-185
Source: J Biomed Sci. 2025 Jun 3;32:55. doi: 10.1186/s12929-025-01149-3 (PMC12131650; doi:10.1186/s12929-025-01149-3)
Supplement: Supplementary file 1 — Additional file 1. [file 12929_2025_1149_MOESM1_ESM.doc]

**Suppl. Table 1.** The demographic data and scores of Updated Sydney System of patients with intestinal metaplasia between probiotic treatment and without.

| Demographic data | Patients with IM | | | *P* value |
| --- | --- | --- | --- | --- |
|  | Probiotic treatment  (n=32) |  | Without treatment  (n=26) |  |
| Age, yr (mean±SD) | 54.5±11.3 |  | 55.6±12.8 | 0.94 |
| Sex=male (%) | 10 |  | 11 | 0.28 |
| Updated Sydney System (mean ±SD) |  |  |  |  |
| Acute inflammatory score | 1.63 ± 0.14 |  | 1.52 ± 0.16 | 0.60 |
| Chronic inflammatory score | 2.87 ± 0.06 |  | 2.77 ± 0.1 | 0.39 |
| *H. pylori* density | 3.23 ± 0.28 |  | 3.4 ± 0.28 | 0.67 |
| Atrophy | 1.7 ± 0.2 |  | 1.72 ± 0.22 | 0.95 |
| Intestinal metaplasia | 2.43 ± 0.12 |  | 2.56 ± 0.14 | 0.50 |

SD: standard deviation.

**Supplement Fig. 1.** The relative quantity of miR-185 in **A** miR-185 mimic cells with differential incubation periods and **B** miR-185 inhibitor cells with various dosages. The **C** phosphorylated β-catenin and **D** COX-2 levels in GES-1 cells transfected with various inhibitor dosages.

**Supplement Fig. 2.** Effect of pretreatment with probiotics (A. *L. acidophilus*, B. *B. lactis*, A+B. mixture of A and B) on **A** nuclear p-STAT3 and **B** COX-2 in AGS cells after *H. pylori* infection.

**Supplement Fig. 3.** The study flow diagram and the number of cases in the different follow-up periods. EGD, esophagogastroduodenoscopy; IM, intestinal metaplasia.

**Supplement Fig. 4.** Different *H. pylori* strains 238, 26695, and 1031 induce nuclear β-catenin and COX-2 expression in GES-1 cell. *H. pylori* incubated with GES-1 cells, the nuclear protein was harvested for **A** β-catenin at 4 hours and **B** COX-2 at 24 hours, respectively.
