# Supplementary figures and images for: Probiotics ameliorate H. pylori-associated gastric β-catenin and COX-2 carcinogenesis signaling by regulating miR-185
Source: J Biomed Sci. 2025 Jun 3;32:55. doi: 10.1186/s12929-025-01149-3 (PMC12131650; doi:10.1186/s12929-025-01149-3)

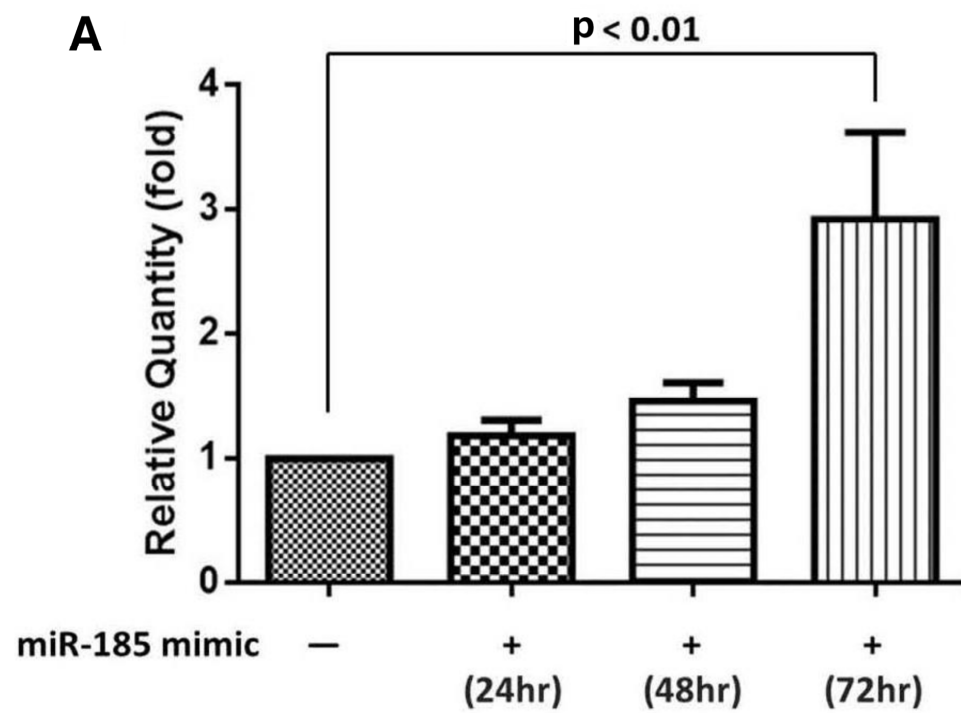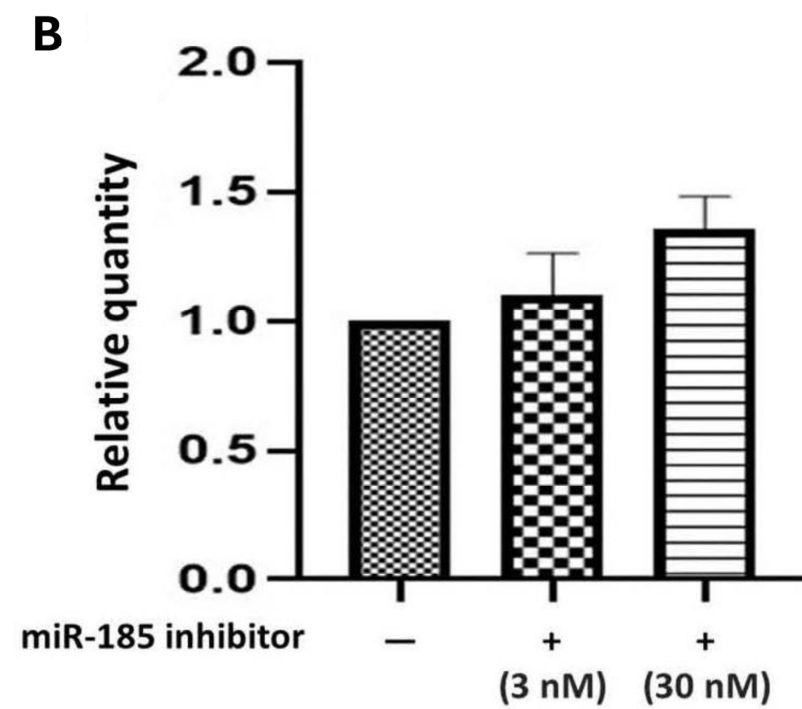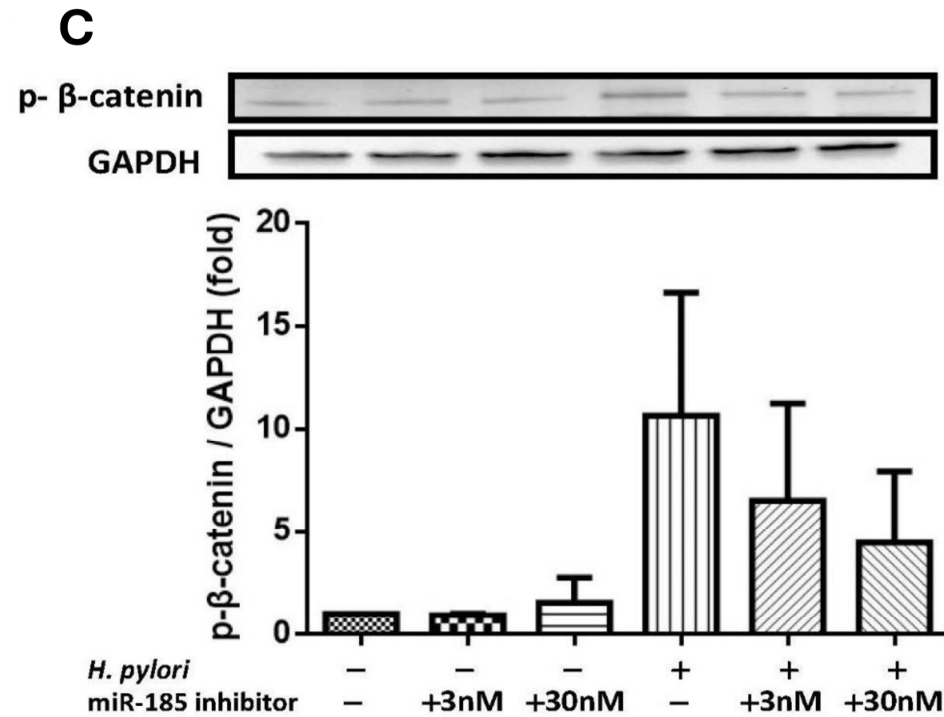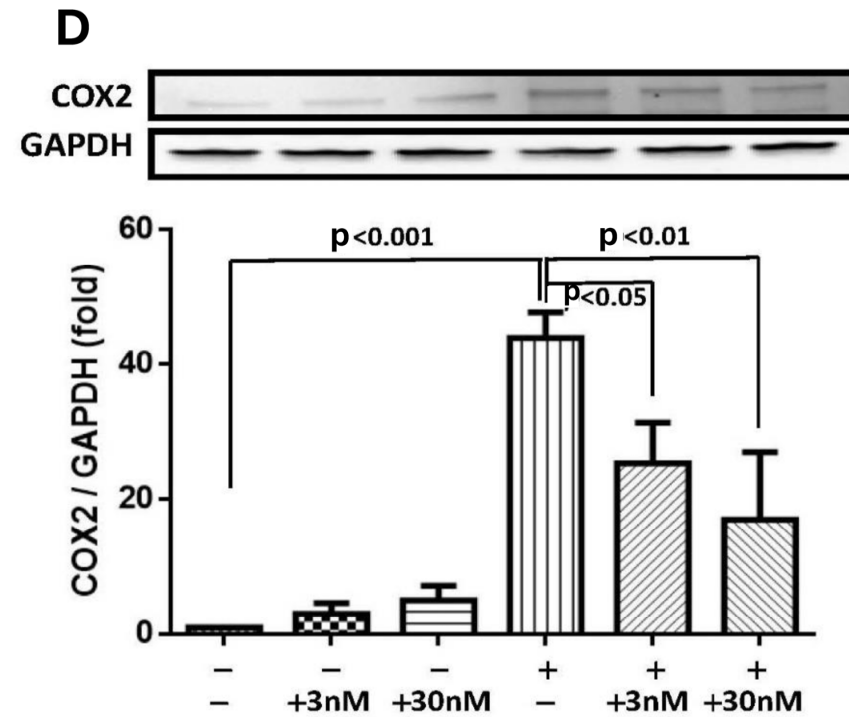

Supplement: Supplementary file 2 — Additional file 2. [file 12929_2025_1149_MOESM2_ESM.pdf]

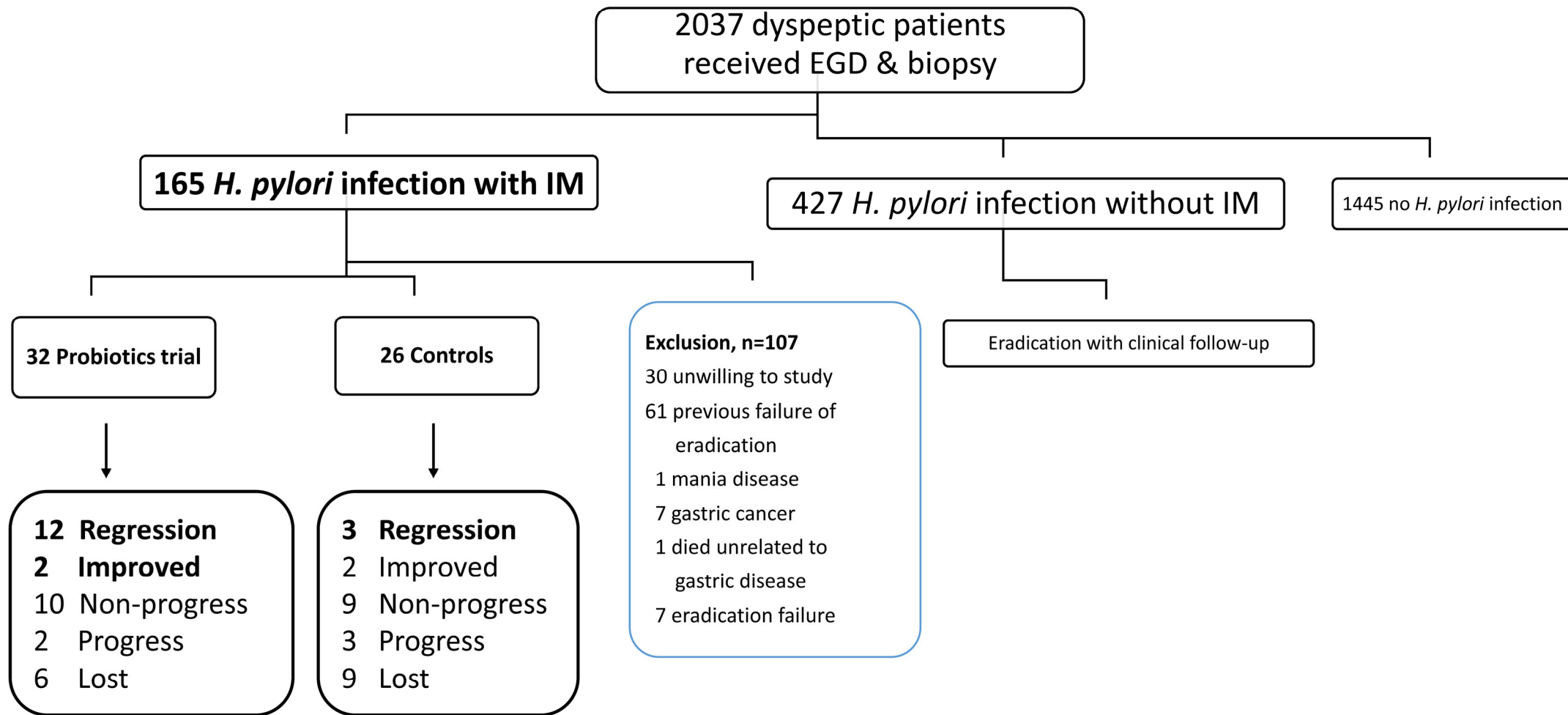

Supplement: Supplementary file 3 — Additional file 3. [file 12929_2025_1149_MOESM3_ESM.pdf]

# AGS

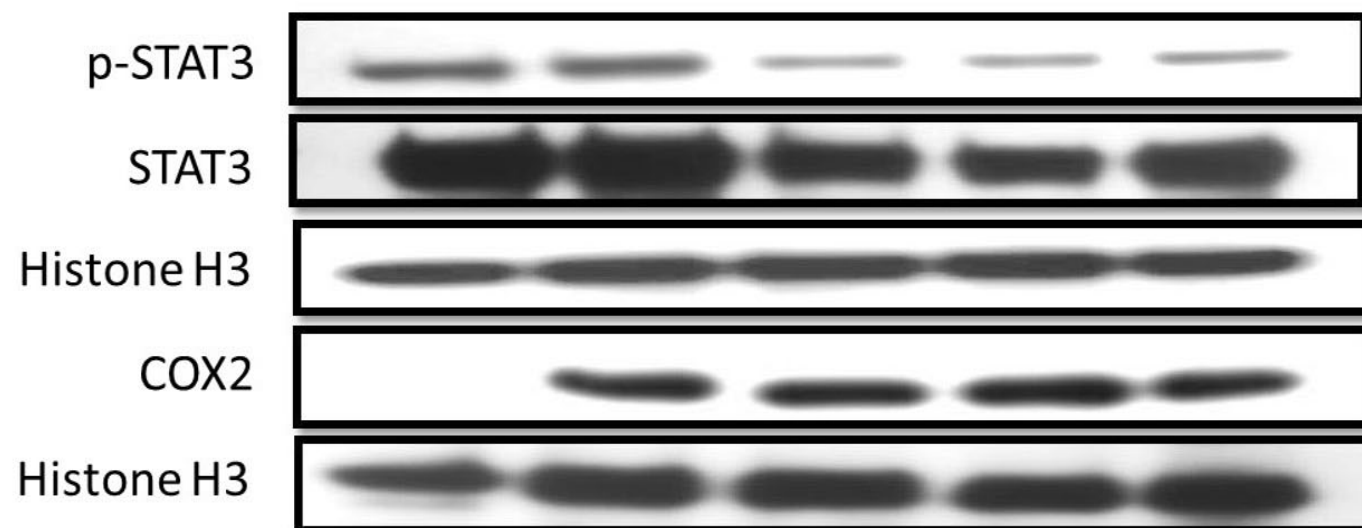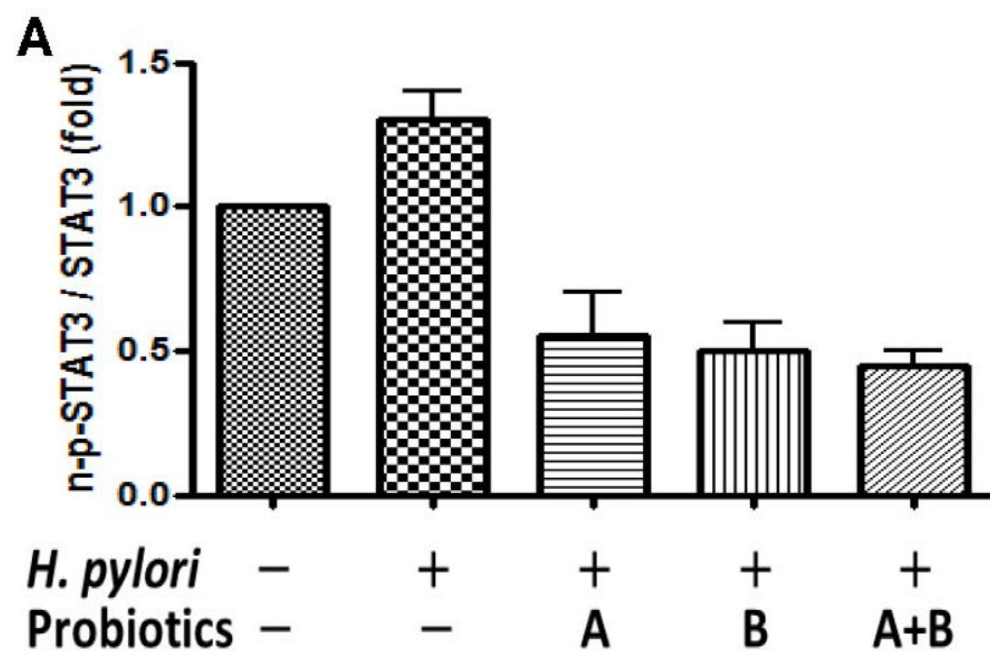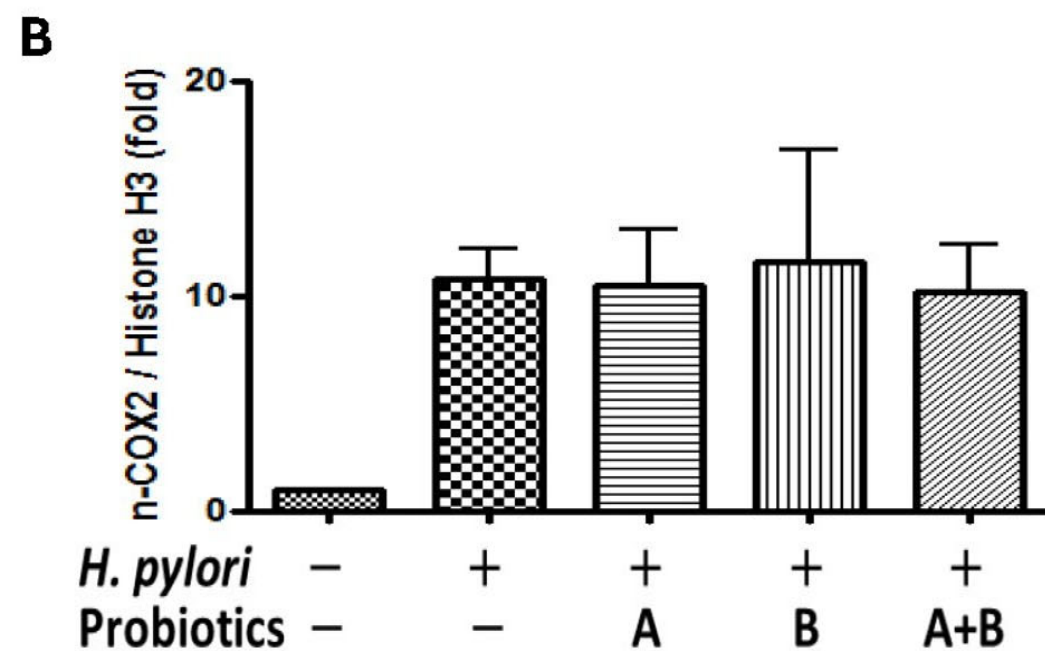

Supplement: Supplementary file 4 — Additional file 4. [file 12929_2025_1149_MOESM4_ESM.pdf]

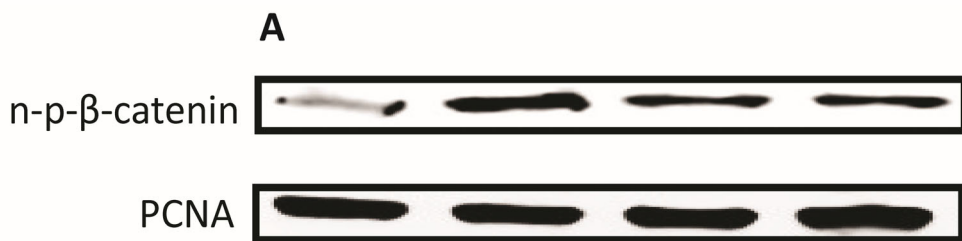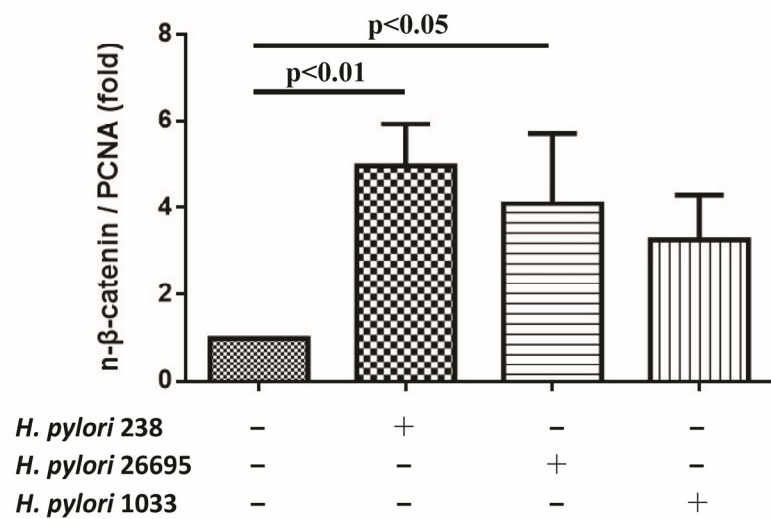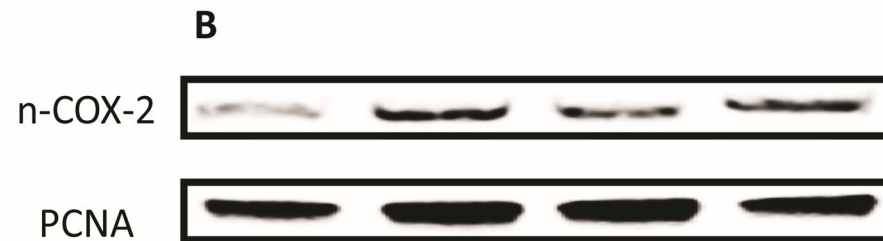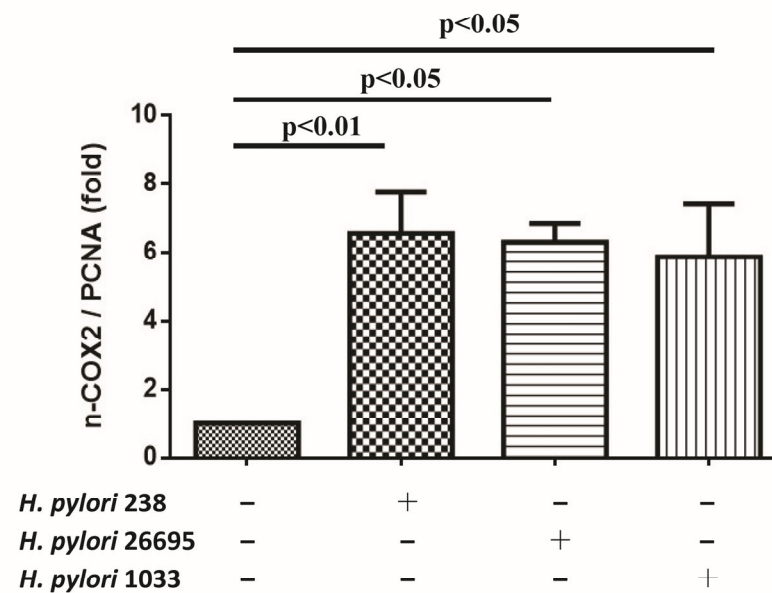

Supplement: Supplementary file 5 — Additional file 5. [file 12929_2025_1149_MOESM5_ESM.pdf]
